# Obstructive lower urinary tract symptoms due to ball valve effect of a rare bladder tumor in an adolescent male

**DOI:** 10.1016/j.radcr.2026.06.101

**Published:** 2026-07-20

**Authors:** Sajad Ahmad Para, Waseem Jan Shah, Zafirah Zahir, Abdul Rouf Khawaja, Uvaisullah Quadir, Gokul K, Omar Mukhtar

**Affiliations:** a Department of Urology, Sher-i-Kashmir Institute of Medical Sciences, Srinagar, India; b Pathology, Sher-i-Kashmir Institute of Medical Sciences, Srinagar, India

**Keywords:** Adolescent bladder tumor, Bladder polyp with ball valve effect, Cystitis glandularis

## Abstract

Cystitis glandularis is a rare inflammatory proliferative disease of urinary bladder. It is commonly associated with chronic bladder outlet obstruction and persistent bladder irritation. It is rarely reported in pediatric population and usually presents with hematuria and lower urinary tract symptoms. Radiological imaging cannot positively differentiate it from more sinister pathologies like bladder malignancies. We report a rare case of bladder outlet obstruction in an adolescent boy caused by the ball-vale effect of a long pedunculated bladder polyp. The case was managed with transurethral resection and histopathology was consistent with cystitis glandularis.

## Introduction

Bladder outlet obstruction (BOO) in children usually presents with poor flow, intermittency, straining or sense of incomplete bladder emptying. Etiology can be either functional or anatomical and can lead to bladder trabeculations, vesicoureteric reflux and in worst cases to obstructive nephropathy. Anatomical causes of BOO in adolescent males can be a neglected posterior urethral valve, urethral calculus, urethral stricture, cecoureterocele, prostatic cysts or prostatic mass. Cystitis glandularis (CG), initially described by Morgagni et al in 1761, is an unusual benign pathology characterized by reactive metaplasia of bladder urothelium [1]. The condition is associated with chronic bladder inflammation or obstruction leading to hyperproliferation of bladder mucosa. It presents with hematuria, irritative lower urinary tract symptoms or rarely with obstructive lower urinary tract symptoms (LUTS) [2]. We present a rare case of bladder outlet obstruction in an adolescent male caused by long-stalk cystitis glandularis bladder polyp producing a ball-valve effect at the posterior urethra.

## Case report

**Fig. 1:**
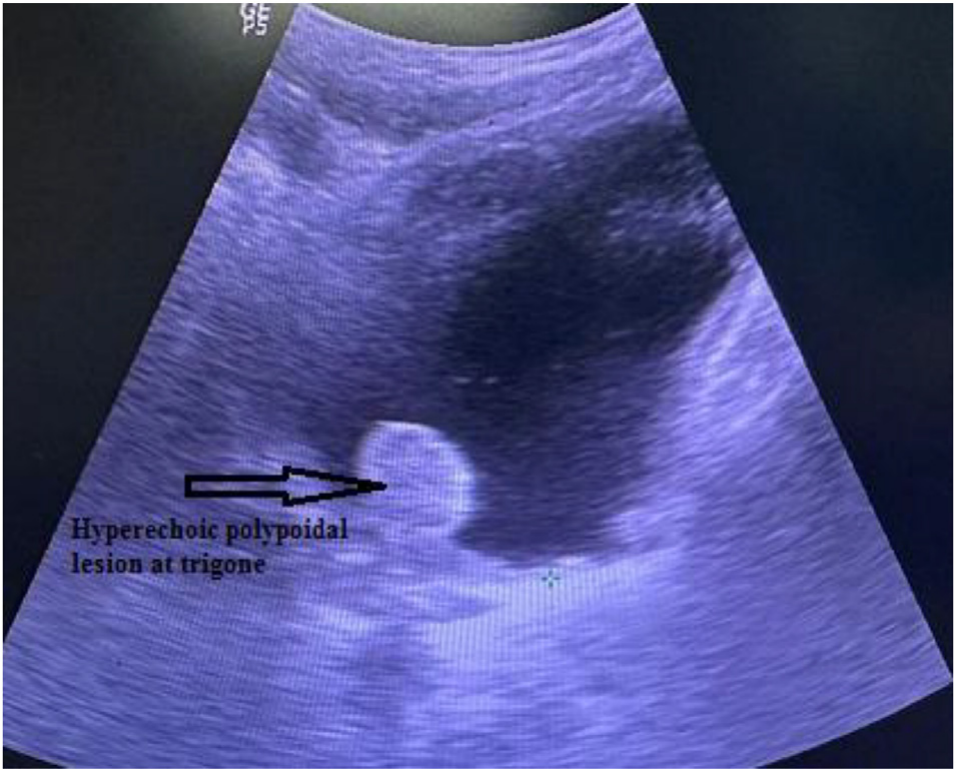
Sagital view with curvilinear ultrasound probe (5htz) showing smooth and rounded bladder mass near bladder neck (black arrow).

**Fig. 2:**
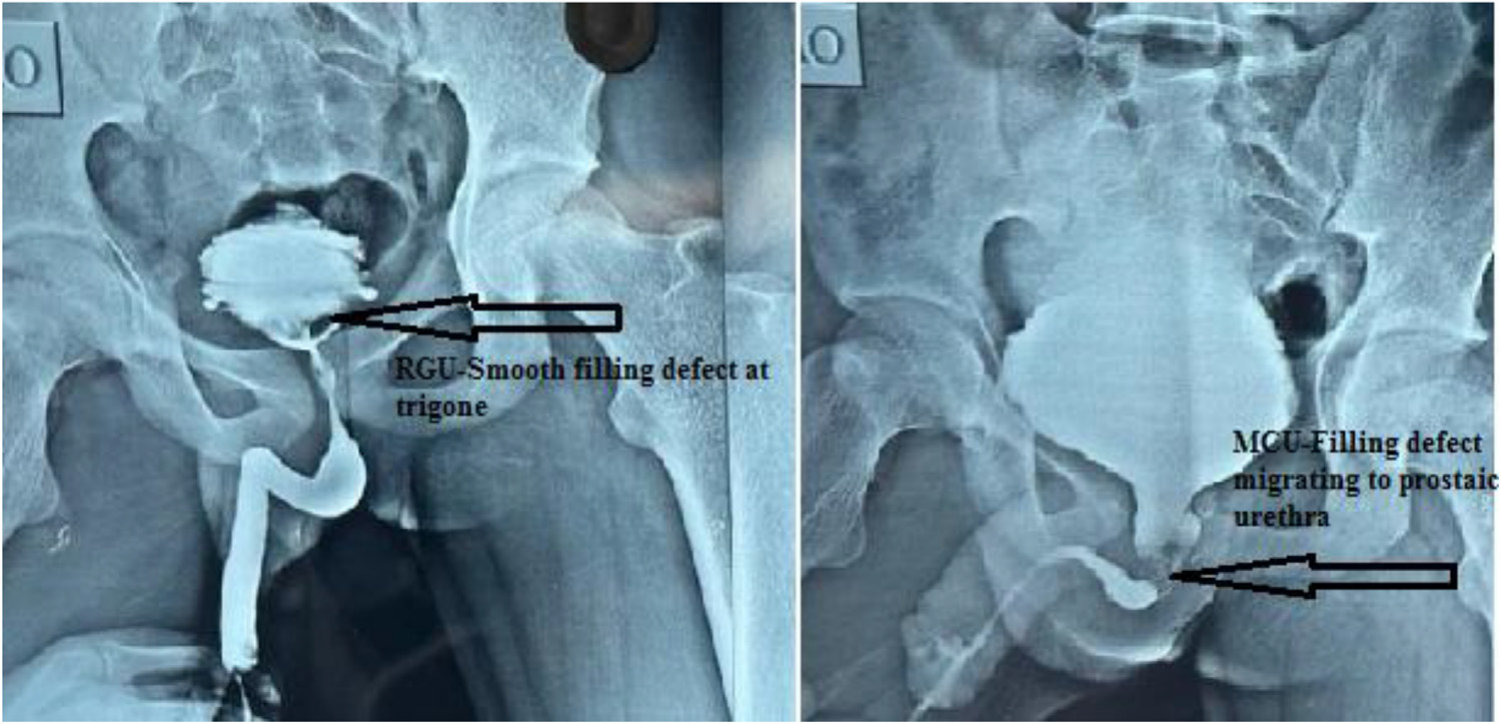
RGU suggestive of filling defect at bladder neck (black arrow) which migrates to posterior urethra on VCU.

**Fig. 3:**
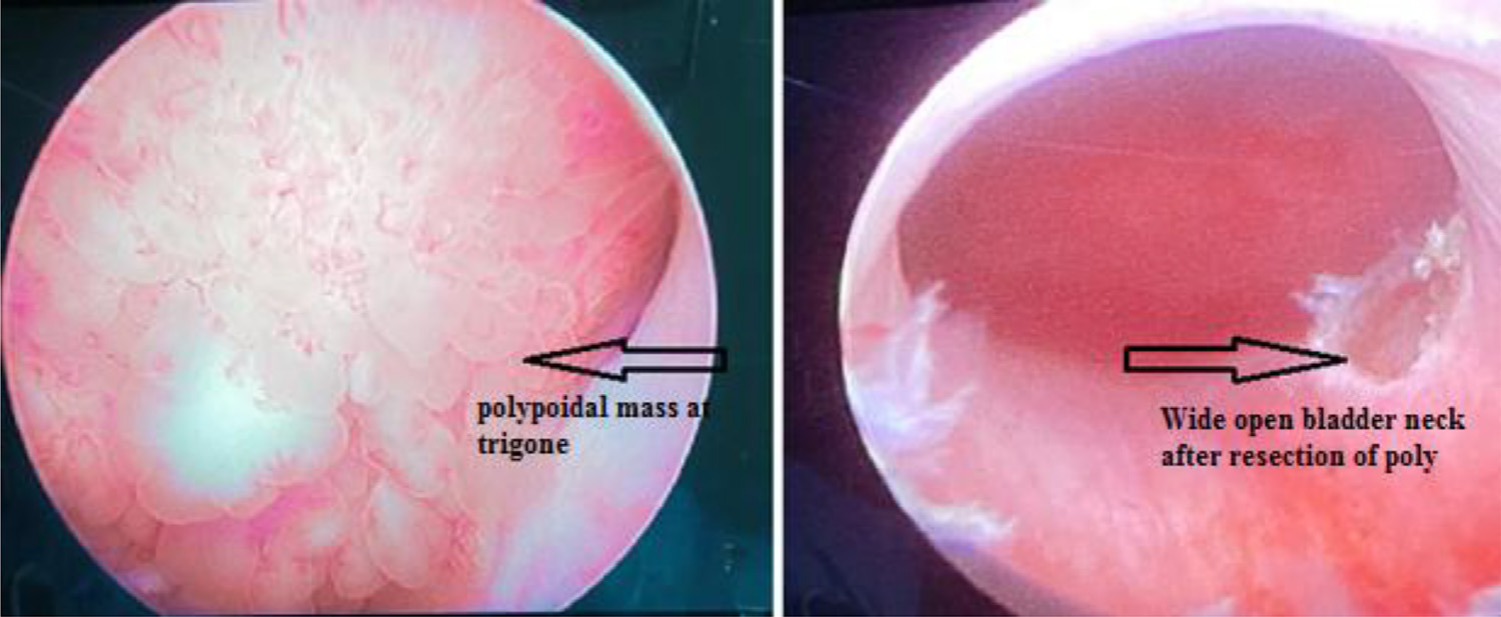
Endoscopic view with 30^0^ rigid lens showing bladder polyp before and after resection (black arrow).

**Fig. 4:**
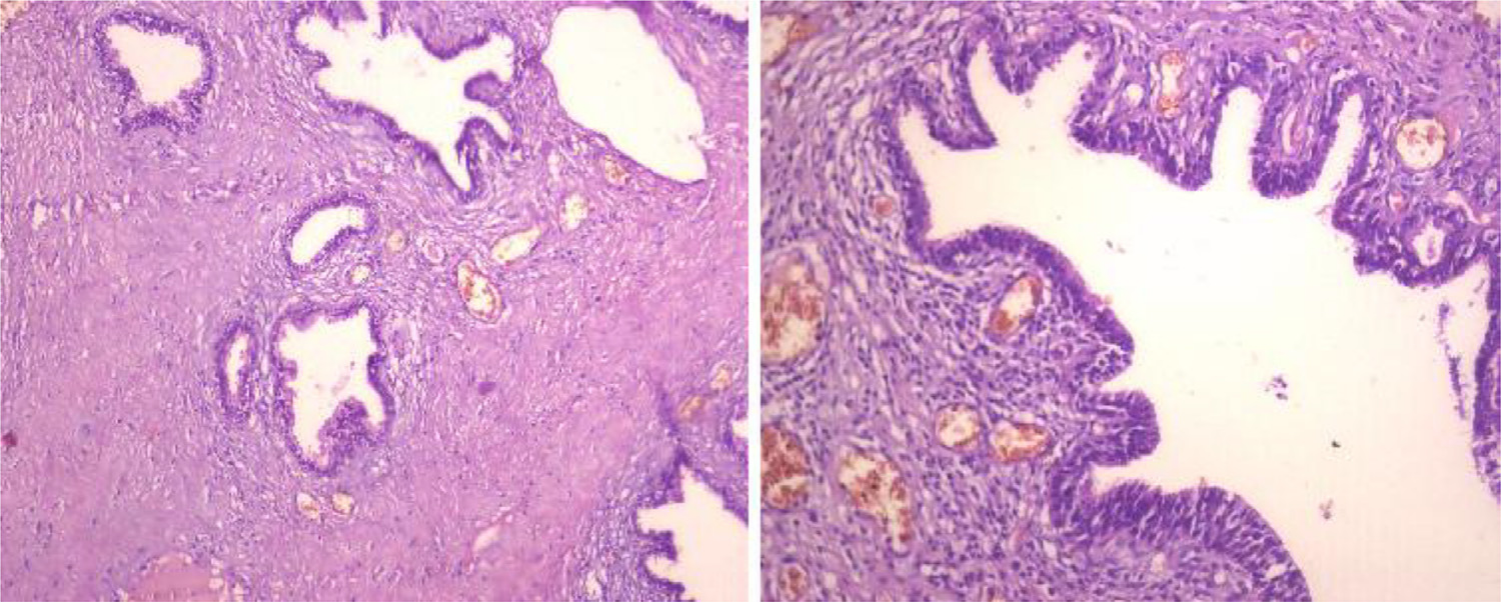
Histopathology showing urothelium with mucosal invaginations, nests of urothelial cells and glands in lamina propria.

A 17 year old boy presented with chief complaints of poor urinary stream, straining, intermittency and overflow incontinence for 2 months. The boy had no prior history of hematuria or lower urinary tract symptoms. Examination revealed a palpable bladder that failed to recede completely following voiding. The external genitalia were normal and neurological examination didn’t reveal any abnormality. The initial ultrasound (USG) examination showed thick walled urinary bladder with significant post-void residual urine. The upper urinary tract was normal however a pedunculated polypoidal mass measuring 3×4cm was documented on the posterior bladder wall near neck (Fig. 1). The color doppler study revealed vascularity within the lesion and demonstrated its descent into prostatic urethra on voiding maneuver. Retrograde urethrography revealed a normal urethra with a smooth filling defect near the bladder neck. On voiding cystourethrography the same filling defect was seen to migrate into the posterior urethra (Fig. 2). Patient was planned for cystoscopic examination under general anesthesia. Cystoscopy revealed a polypoidal growth (4×3cm) with long stalk arising from the bladder neck on left side and completely blocking the bladder neck. A 16Fr non continuous resectoscope was introduced and the polyp was resected piecemeal with Monopolar cutting electrode (Fig. 3). Procedure was uneventful and patient had complete resolution of symptoms. Histopathology revealed polypoidal structures with broad based non-branching fronds lined by benign urothelium. The urothelium showed mucosal invaginations, nests of urothelial cells and glands in lamina propria consistent with cystitis glandularis (Fig. 4).

## Discussion

Cystitis glandularis is the benign proliferative disorder of the bladder mucosa arising in the background of chronic bladder outlet obstruction or bladder irritation. The etiopathogenesis of this rare bladder tumor remains poorly understood with some believing it to be related to abnormal embryonic migration of intestinal germ cells from rectum while as others attribute it to metaplastic transformation in response to chromic irritation [3]. Immune mechanism involvement had been suggested by many researchers citing the abnormally high level of IgA on the surface of urothelial cells in CG [4]. Most common clinical presentation of CG is hematuria and irritative lower urinary tract symptoms. In advanced cases and trigonal involvement can lead to obstructive uropathy Our patient presented with obstructive LUTS and overflow incontinence due to pedunculated bladder polyp at bladder neck. On voiding the lesion popped down into the posterior urethra leading to poor stream, intermittency and incomplete voiding.

Every bladder lesion is initially evaluated by ultrasound (USG) and CG usually appears as broad based solid lesion involving the trigonal region. Besides that, the status of the upper tract could be simultaneously ascertained. In our case a smooth polypoidal lesion could be easily demonstrated arising from the bladder neck area. Contrast enhanced computed tomography (CECT) is the ideal imaging modality for staging bladder masses. It has a high sensitivity (79-93%) and specificity (92-99%) for detecting bladder lesions. Its utility is limited by its poor sensitivity to detect small flat lesions and inability to positively differentiate CG from malignant bladder pathologies [5]. Hua H et al in their study on bladder cancer and CG concluded that the enhancement CT values and relative enhancement CT values in arterial, venous, and delayed phases were significantly higher in Bladder cancer compared to CG [6]. Differential diagnosis of a bladder mass includes urothelial carcinoma, adenocarcinoma, papillary urothelial neoplasm of low malignant potential (PUNLMP), leomyoma, and rarely paraganglioma. Urothelial cancers appear as enhancing nodular or pedunculated intraluminal masses. Urachal adenocarcinoma typically presents as heterogeneous solid midline masses on anterior bladder wall with low attenuation (mucinous) components. PUNLMP appear as small pedunculated masses that present as soft tissue intraluminal filling defects and are often missed on routine CT. Bladder leomyoma are displayed as smooth hypo or isodense intramural nodules with mild to moderate homogeneous enhancement. Bladder paragangliomas are rare and appear on CT as sharply marginated, hyperdense intramural masses with avid heterogeneous enhancement during the arterial phase, and progressive washout in delayed series [7]. On MR imaging, bladder paragangliomas exhibit marked hyperintensity in both T1 and T2 weighted images. In contrast MRI, they show avid and homogenous enhancement. They exhibit diffusion restriction, appearing hyperintense on DWI and hypointense on ADC maps. Multiparametric Magnetic resonance imaging (mpMRI) which employs sequences like diffusion-weighted (DWI) and dynamic contrast enhanced imaging (DWI) has proved superior to other imaging modalities in assessing depth of tumor infiltration in bladder cancer. However it fails to differentiate the CG from more sinister pathologies like urothelial cancer and benign bladder pathologies like PUNLMP. On MRI, CG usually manifest as nodules or focal thickening in trigonal region. They display slight hyperintensity on T2 weighted images and mild hypointensity on T1 weighted images. There is a progressive enhancement pattern without significant restriction on diffusion weighted images [8]. On cystoscopy CG appears as broad based solid lesion with think fronds usually involving trigone. It is usually performed at the time of endoscopic resection to document size and distribution of disease within bladder [9].

Transurethral resection serves the diagnostic and therapeutic purpose in most of the cases. Both Monopolar and bipolar resecting loops are effective in endoscopic resection with the latter providing smoother cutting, less bleeding and better vision [10]. We used 16Fr monopolar resectoscope for transurethral resection. The boy reported resolution of symptoms and there was no recurrence at three months on USG. Ahmad kusumaputra et al reported a similar case of juvenile cystitis glandularis presenting with obstructive LUTS and hematuria which was managed by endoscopic resection [11]. In our case the mass was pedunculated and lead to obstructive LUTS due to ball valve effect without any hematuria. CG is the microscopic diagnosis characterized by imagination of urothelium into lamina propria subsequently developing into fluid containing vesicles distributed in a more glandular pattern with abundance of cuboidal or columnar cells and mucin producing goblet cells. It may be associated with either intestinal type or glandular metaplasia. Histologically CG has been divided into two sub types, typical cystitis glandularis-lined by mucinous cells and cystitis glandularis intestinal type-lined by intestinal metaplastic cells in addition to goblet cells [12]. Intestinal type of CG, when extensive, is difficult to differentiate from well differentiated adenocarcinoma and actually has a malignant potential. Extensive ectopic cell presence and invasion of muscularis propria in adenocarcinoma differentiates it from intestinal type CG. Extensive CG has high chances of recurrence and patients need close follow up to detect recurrence and associated complications. Both steroids and cyclooxygenase inhibitors-2 (COX-2) has been shown to improve symptoms and reduce recurrence in extensive disease [13]. Intravesical therapy with sodium hyaluronate has been shown to reduce inflammation and provide symptomatic relief. It is given intravesical as 40mg in 50 ml saline weekly till symptomatic relief [14]. We did not offer any medical therapy to our patient because of solitary polypoidal disease and apparently normal bladder.

## Conclusion

Cystitis glandularis is a rare inflammatory proliferative disease of urinary bladder presenting as bladder mass. Incidence is rare in adolescence and clinical presentation is varied. Transurethral resection fulfills diagnostic and therapeutic goals. Small size resectoscopes are pivotal in resecting without traumatizing fragile juvenile urethra.

## Ethical approval

Ethical committee clearance was taken from the institution ethical committee with IEC No. CR#321/25. The work is approved by the institution ethical committee with protocol number of IEC/SKIMS EC331/2026.

## Patient consent

Written informed consent was taken from the above mentioned patient for publishing the data.
